# Supplementary material for: Raising an Eye at Facial Muscle Morphology in Canids
Source: Biology (Basel). 2024 Apr 25;13(5):290. doi: 10.3390/biology13050290 (PMC11118188; doi:10.3390/biology13050290)
Supplement: Supplementary file 1 [file biology-13-00290-s001.zip › biology-2931694-supplementary.pdf]

Supplemental Figures

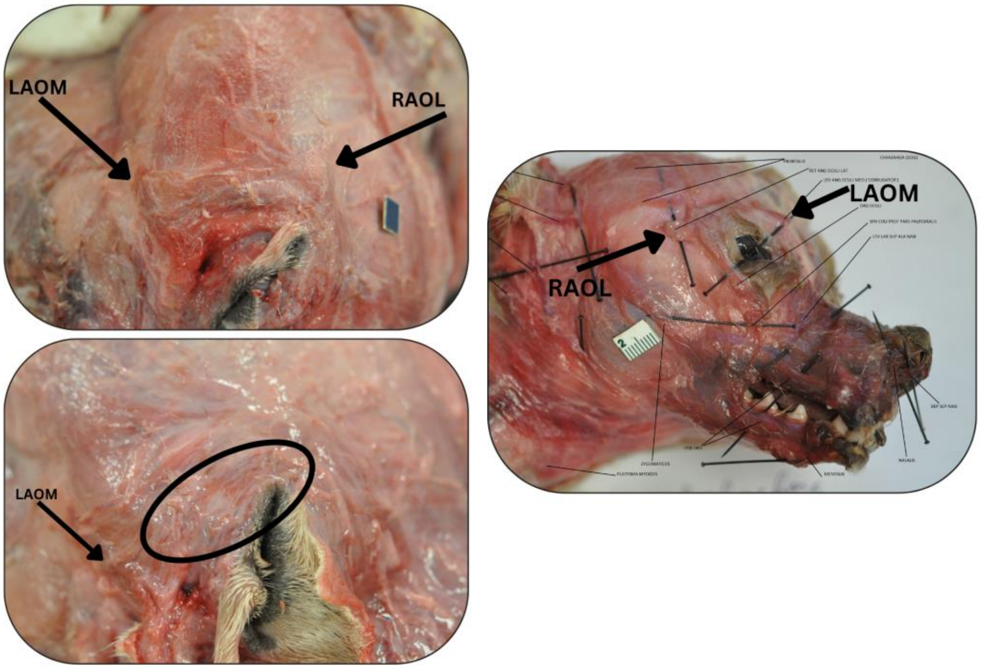

Figure S1: Specimen 1 – Dog (*C. familiaris*) adult/M (right); and Specimen 2 – Dog (*C. familiaris*) adult/M (top left) and detail of striated muscles on Specimen 2 (bottom left).

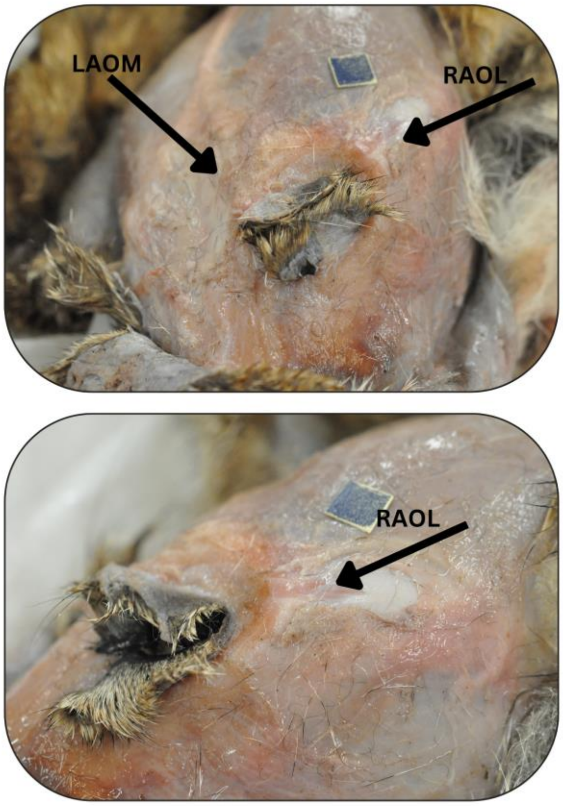

Figure S2: Specimen 3 – Coyote (*C. latrans*) young adult/F.

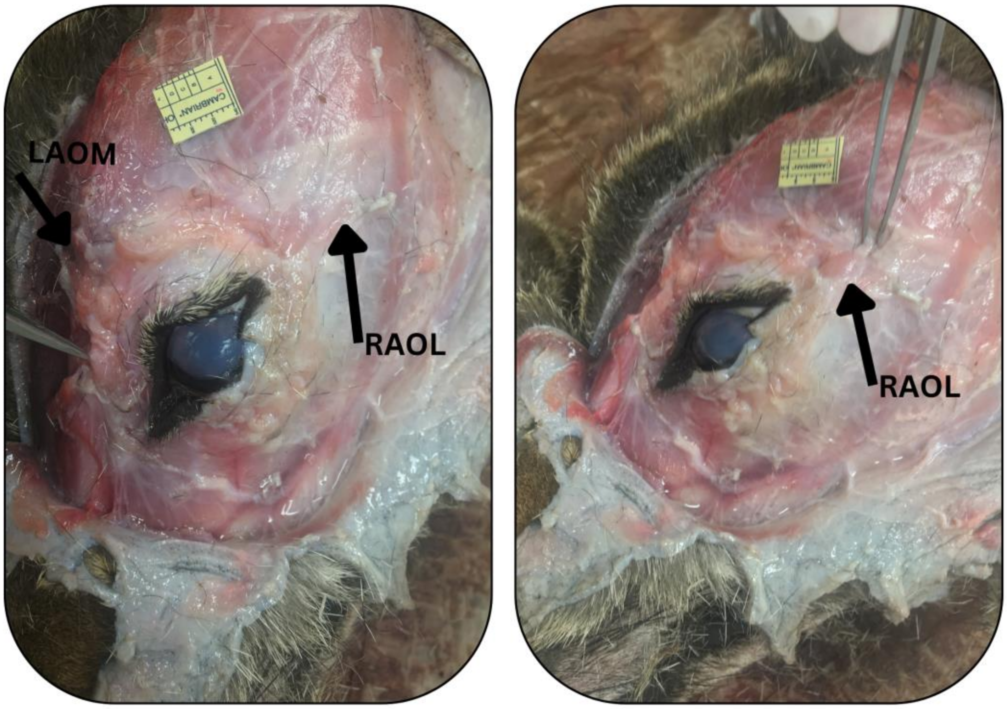

Figure S3: Specimen 4 – Coyote (*C. latrans*) adult/F.

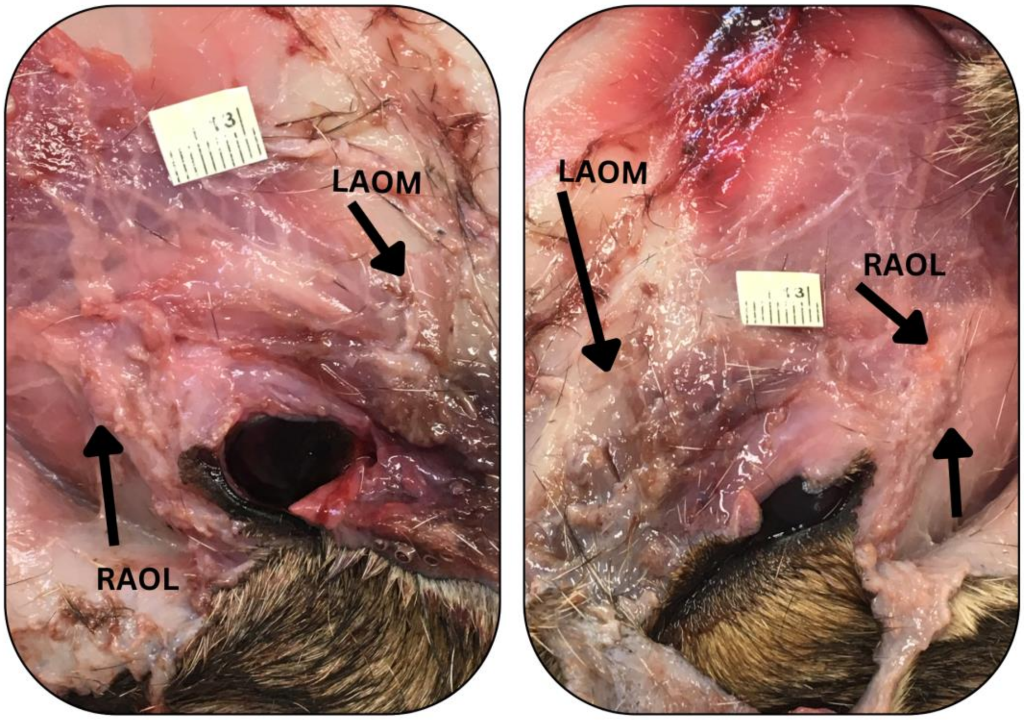

Figure S4: Specimen 5 – Coyote (*C. latrans*) adult/M.

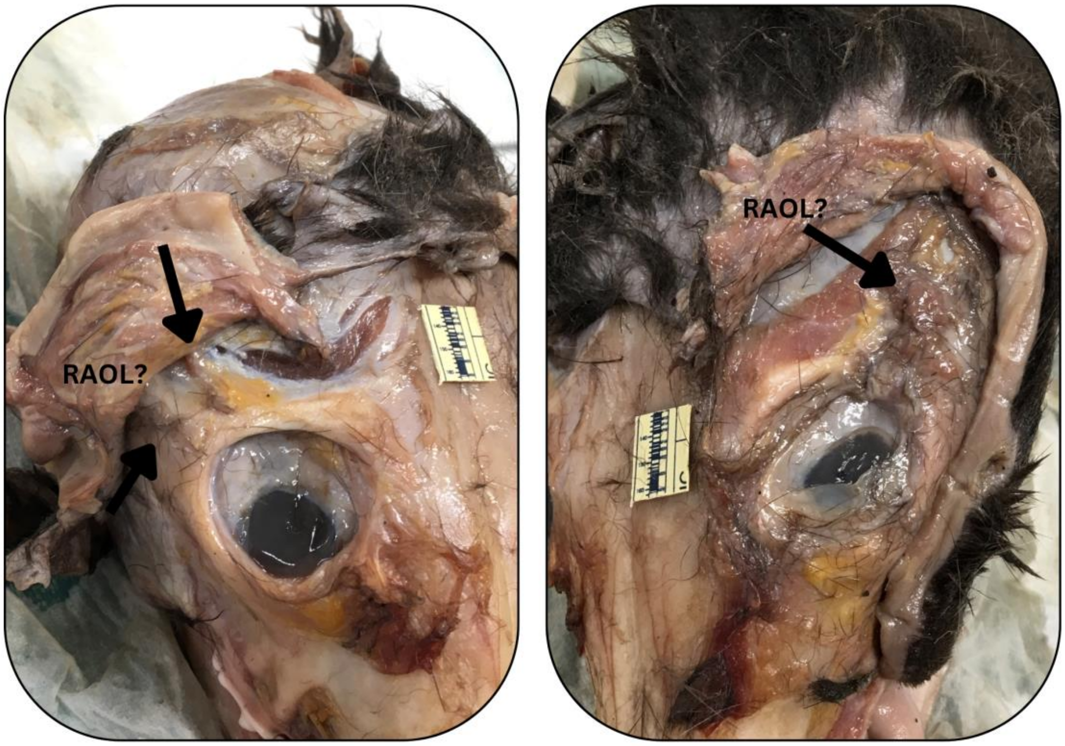

Figure S5: Specimen 6 – Arctic fox (*V. lagopus*) unk/unk.

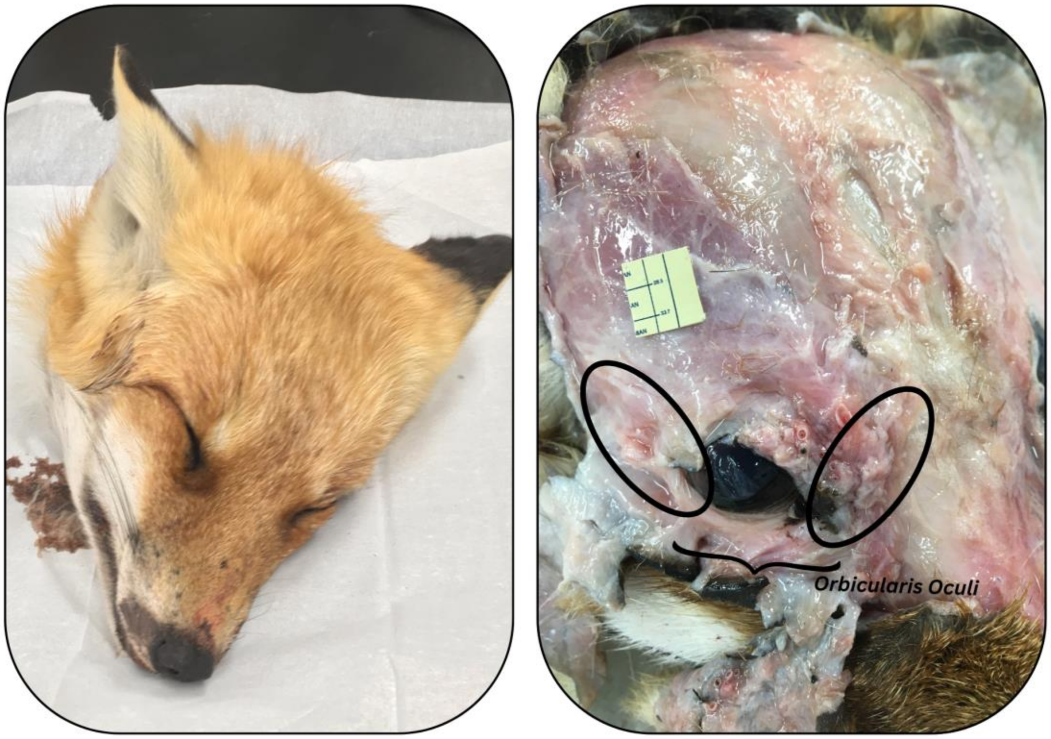

Figure S6: Specimen 7 – Red fox (*V. v. fulvus*) adult/F – the possible *retractor* seems indistinct from the *orbicularis*, as does the *levator* homolog.

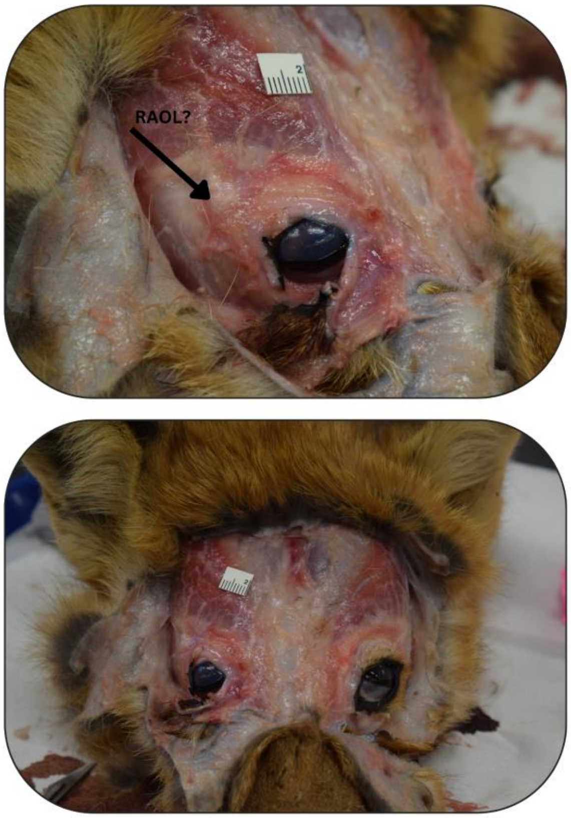

Figure S7: Specimen 8 – Red fox (*V. v. fulvus*) juvenile/M.

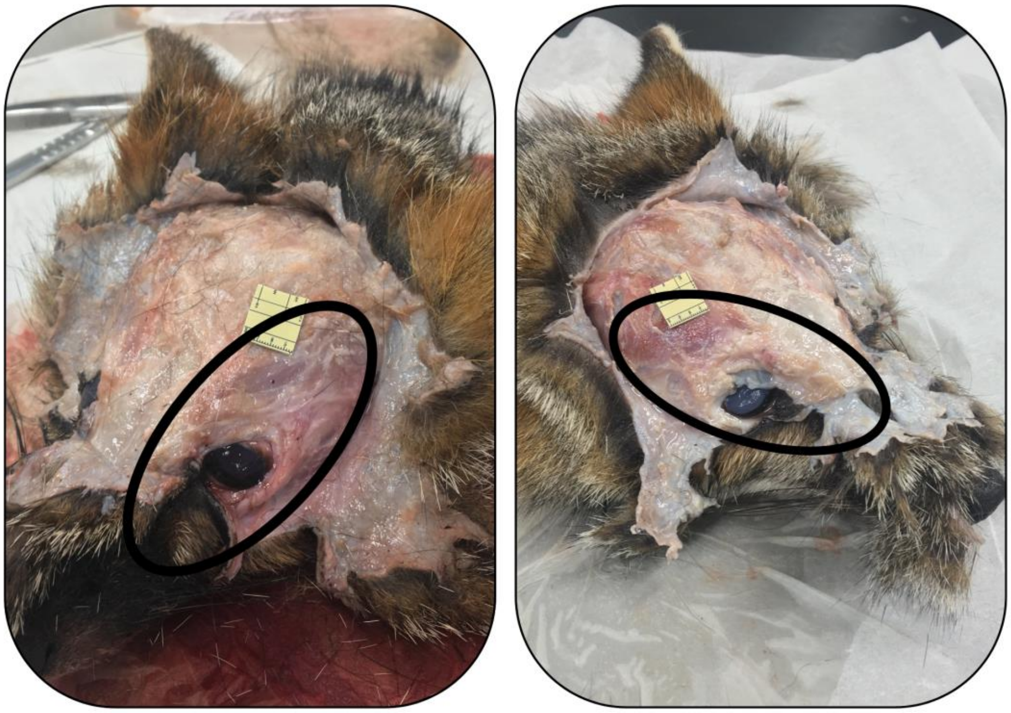

Figure S8: Specimen 9 – Gray fox (*U. cinereoargenteus*) adult/F (left); and Specimen 10 – Gray fox (*U. cinereoargenteus*) adult/M (right).
